# Complete Genome Sequence of the Extensively Drug-Resistant Extended-Spectrum β-Lactamase-Producing Proteus mirabilis Isolate HK294, Obtained from Poultry Feces in Hong Kong

**DOI:** 10.1128/mra.00225-23

**Published:** 2023-05-22

**Authors:** Michael Biggel, Sara Boss, Theethawat Uea-Anuwong, Kittitat Lugsomya, Ioannis Magouras, Roger Stephan

**Affiliations:** a Institute for Food Safety and Hygiene, Vetsuisse Faculty, University of Zürich, Zürich, Switzerland; b Department of Infectious Diseases and Public Health, Jockey Club College of Veterinary Medicine, City University of Hong Kong, Hong Kong SAR, China; c Veterinary Public Health Institute, Department of Clinical Research and Veterinary Public Health, Vetsuisse Faculty, University of Bern, Bern, Switzerland; University of Maryland School of Medicine

## Abstract

Here, we report the complete genome sequence of Proteus mirabilis isolate HK294, recovered from pooled poultry feces in Hong Kong in 2022. The chromosome contained 32 antimicrobial resistance genes, including the extended-spectrum β-lactamases *bla*_CTX-M-65_ and *bla*_CTX-M-3_. Almost all resistance genes were part of either an integrative conjugative element or a Tn*7*-like transposon.

## ANNOUNCEMENT

Proteus mirabilis is a frequent cause of nosocomial infections and widely distributed throughout the natural environment and intestines of humans and animals. P. mirabilis is naturally resistant to various antimicrobial drugs but is usually sensitive to ampicillin, cephalosporins, and aminoglycosides (1, 2).

HK294 was isolated in June 2022 from a pooled fecal sample from a poultry flock in Hong Kong. The sample (0.3 g) was enriched in 2.7 mL brain heart infusion (BHI) broth (BD) with cefotaxime (3.5 μg/mL) and vancomycin (32 μg/mL) at 37°C for 24 h. One loopful of the broth was spread on Brilliance extended-spectrum β-lactamase (ESBL) agar (Oxoid) and incubated at 37°C for 24 h. Matrix-assisted laser desorption ionization–time of flight mass spectrometry (MALDI-TOF MS; Bruker Daltonics) was used for species identification. Disk diffusion susceptibility testing was performed for 16 antimicrobial agents and interpreted according to CLSI protocols (3).

DNA was isolated from subcultures obtained from single colonies grown for 24 h at 37°C on sheep blood agar. For short-read sequencing, genomic DNA was extracted using the DNeasy blood and tissue kit (Qiagen). Libraries were prepared using the Nextera DNA Flex library preparation kit (Illumina) and sequenced on the Illumina MiniSeq platform (2 × 150 bp). Read trimming and quality control were performed using fastp v0.20.1 (4). For long-read sequencing, DNA was extracted using the MasterPure kit (Lucigen) (no size selection/shearing). Libraries prepared using the SQK-LSK112 and EXP-NBD114 barcoding kits were sequenced on a MinION FLO-MIN112 flow cell (Oxford Nanopore). Base calling was performed using Guppy v6.1.1 (https://community.nanoporetech.com) and quality assessed using nanoq v0.9.0 (5). A hybrid assembly was generated from 373 Mbp short-read (1,254,71x1 paired reads; coverage, 93×) and 105 Mbp long-read data (15,165 reads; read *N*_50_, 23 kb; coverage, 20×) using the Unicycler v0.5 pipeline (6), which includes circularization and rotation. The assembly was annotated using PGAP v2022-12-13 (7). Resistance genes were detected using AMRFinder v3.10.24 (8).

**FIG 1:**
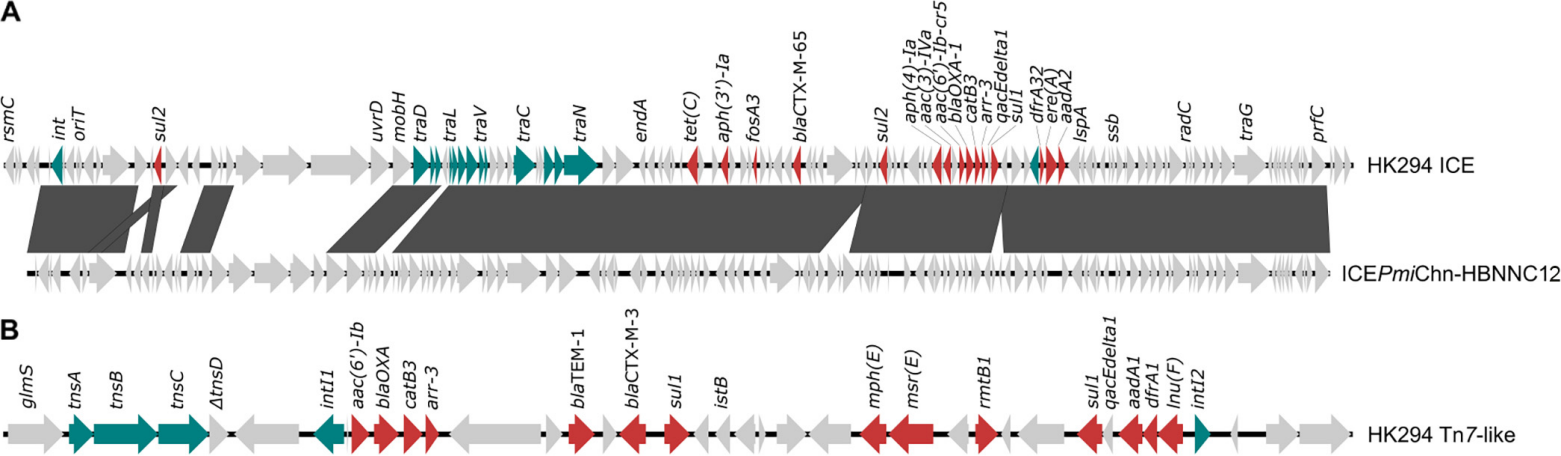
Genetic environment of antimicrobial resistance genes identified in the chromosome of HK294. (A) Comparison of the *bla*_CTX-M-65_-containing region in HK294 to the integrative conjugative element ICE*Pmi*Chn-HBNNC12 (GenBank accession number MZ277865.1). Gray shading indicates homologous regions. (B) Structure of the Tn*7*-like transposon harboring *bla*_CTX-M-3_. Antimicrobial resistance genes are shown in red. Integrases, *tra* genes, and the transposition genes *tnsABC* are shown in teal. The figures were generated using Easyfig v2.1 (10).

The genome of HK294 consisted of a 4,003,686-bp chromosome (GC content, 39.1%) and contained 27 distinct resistance genes, some (*arr-3*, *catB3*, *sul1*, and *sul2*) in more than one copy. Most (31/32) resistance genes were on a putative integrative conjugative element (ICE) or a Tn*7*-like transposon. The putative ICE contained *aac(6')-Ib-cr5* (fluoroquinolone/aminoglycoside resistance), *arr-3* (rifamycin resistance), *bla*_CTX-M-65_ (cephalosporin resistance), and *fosA3* (fosfomycin resistance), among others (Fig. 1A). It further contained an origin of transfer and transfer (*tra*) genes. An NCBI BLASTn analysis (nucleotide sequence collection) (9) revealed high homology to ICE*Pmi*Chn-HBNNC12 (GenBank accession number MZ277865.1).

The Tn*7*-like transposon was integrated downstream of *glmS* and comprised the transposase genes *tnsABC* and a nonfunctional class 2 integron integrase (*intI2*) at the opposite end. The transposon contained *bla*_CTX-M-3_ (cephalosporin resistance), *lnu*(F) (lincosamide resistance), and *mph*(E) and *msr*(E) (both conferring macrolide resistance), among others (Fig. 1B). HK294 was resistant to azithromycin (macrolide), ampicillin and cefazolin (β-lactams), nalidixic acid, ciprofloxacin (fluoroquinolone), streptomycin, kanamycin, and gentamicin (aminoglycosides), chloramphenicol, tetracycline, trimethoprim-sulfamethoxazole, and fosfomycin. P. mirabilis resides in the intestinal tract of chickens. Fecal contamination during slaughter may hence be a transmission route of multidrug-resistant P. mirabilis to the consumer.

### Data availability.

Sequencing data were deposited in the NCBI Sequence Read Archive (SRA) under accession numbers SRR23693645 (short reads) and SRR23693644 (long reads) and BioProject accession number PRJNA935533. The assembly is available under GenBank accession number GCA_029201285.1.
